# Evaluation of suitable reference genes for gene expression studies in porcine alveolar macrophages in response to LPS and LTA

**DOI:** 10.1186/1756-0500-5-107

**Published:** 2012-02-18

**Authors:** Mehmet Ulas Cinar, Mohammad Ariful Islam, Muhammad Jasim Uddin, Ernst Tholen, Dawit Tesfaye, Christian Looft, Karl Schellander

**Affiliations:** 1 Institute of Animal Sciences, Unit of Animal Breeding and Husbandry, University of Bonn, Endenicher Allee 15, 53115 Bonn, Germany; 2 Department of Medicine, Faculty of Veterinary Science, Bangladesh Agricultural University, Mymensing 2202, Bangladesh

**Keywords:** Candidate reference genes, Alveolar macrophage, LPS, LTA, Pigs

## Abstract

**Background:**

To obtain reliable quantitative real-time PCR data, normalization relative to stable housekeeping genes (HKGs) is required. However, in practice, expression levels of 'typical' housekeeping genes have been found to vary between tissues and under different experimental conditions. To date, validation studies of reference genes in pigs are relatively rare and have never been performed in porcine alveolar macrophages (AMs). In this study, expression stability of putative housekeeping genes were identified in the porcine AMs in response to the stimulation with two pathogen-associated molecular patterns (PAMPs) lipopolysaccharide (LPS) and lipoteichoic acid (LTA). Three different algorithms (geNorm, Normfinder and BestKeeper) were applied to assess the stability of HKGs.

**Results:**

The mRNA expression stability of nine commonly used reference genes (*B2M, BLM, GAPDH, HPRT1, PPIA, RPL4, SDHA, TBP *and *YWHAZ*) was determined by qRT-PCR in AMs that were stimulated by LPS and LTA *in vitro*. mRNA expression levels of all genes were found to be affected by the type of stimulation and duration of the stimulation (*P *< 0.0001). geNorm software revealed that *SDHA, B2M *and *RPL4 *showed a high expression stability in the irrespective to the stimulation group, while *SDHA, YWHAZ *and *RPL4 *showed high stability in non-stimulated control group. In all cases, *GAPDH *showed the least stability in geNorm. NormFinder revealed that *SDHA *was the most stable gene in all the groups. Moreover, geNorm software suggested that the geometric mean of the three most stable genes would be the suitable combination for accurate normalization of gene expression study.

**Conclusions:**

There was discrepancy in the ranking order of reference genes obtained by different analysing algorithms. In conclusion, the geometric mean of the *SDHA, YWHAZ *and *RPL4 *seemed to be the most appropriate combination of HKGs for accurate normalization of gene expression data in porcine AMs without knowing the type of bacterial pathogenic status of the animals.

## Background

Alveolar macrophages (AMs) are thought to be critical in the pathogenesis of several lung diseases [1]. Swine respiratory diseases, which has been described world-wide, affects swine of all ages and has a serious impact on economy, ecology and animal welfare in the pig rearing industry [2]. Both Gram-positive and Gram-negative bacteria are causing respiratory disease in pigs [3]. As an *in vitro *model for the development of lung inflammation, AMs stimulation with PAMPs in culture is being frequently used for immunogenetic research in pigs [4-7]. Lipopolysaccharide (LPS) and lipoteichoic acid (LTA) are the PAMPs of the Gram-negative and the Gram-positive bacterial cell wall that cause activation of an acute inflammatory response *in vitro *as well as *in vivo*. Gene expression assay is a common way to investigate the defensive role of AMs in the bacterial infections as well as to dissect the pathogenesis of bacterial lung diseases. With this purposes, several studies focusing on gene expressions have been conducted in AMs *in vitro *[4-7]. The gene expression are required to normalize for housekeeping genes (HKGs) which have tremendous effect on the results of expression study [8]. Therefore, it is crucial to know whether the expression stability of HKGs in AMs is affected by various PAMPs from infectious agents but these data are currently unavailable for pigs.

Quantitative real-time PCR (qRT-PCR) is a powerful technique for gene expression studies, which have become increasingly important in a large number of clinical and scientific fields [8,9]. Besides being a powerful technique, inappropriate data normalization is the most important problem in qRT-PCR [8]. For an exact comparison of mRNA transcription in different samples or tissues, it is crucial to choose the appropriate reference gene [9]. The most accepted approach to mRNA quantification is normalization of the expression level of a gene of interest (target gene) to the expression level of a stably expressed internal reference gene [8,9]. Normalizing to a reference gene is a widely used method because it is simple in theory. Normalizing to a single reference gene is often used but Vandesompele et al. [8] suggested that geometric mean of multiple carefully selected HKGs is recommendable and suitable for accurate normalization. The normalization adjusts for differences in the quality or quantity of template RNA or starting material and differences in RNA preparation and cDNA synthesis, since the reference gene is exposed to the same preparation steps as the gene of interest. This allows the direct comparison of normalized transcript expression levels between samples. Reference genes should ideally be constitutively expressed by all cell types and should not be affected by disease and experimental procedure. To date, a universal reference gene has not been identified. HKGs are most commonly used reference genes [8]. Although HKGs are expressed by any cell, their expression varies among different cell types/organs, age, sex and treatment or experimental conditions [10-17]. Use of HKGs as reference genes for a particular sample type should be, therefore, validated.

Ideally, the conditions of the experiment should not influence the expression of the reference genes [18]. However, the mRNA expression of reference genes from different cells and tissues [18-21] such as from AMs [1,10] may fluctuate due to infectious agents *in vitro*. Alveolar macrophages are being used as an important model to dissect the pathogenesis and genetics behind the infection through gene expression studies [5,6,22,23]. To date, no reference genes have been validated for expression studies of AMs in pigs. The aim of this study was therefore to identify a set of stably expressed reference genes in porcine AMs cells irrespective of stimulation as well as in the case of stimulation by bacterial LTA and LPS *in vitro*.

## Methods

### Animals and preparation of alveolar macrophage cells

Fourty-day-old three German Landrace piglets were euthanized for sampling. All animals were healthy and exhibited no signs of hypoxia or asphyxia or infections. Animals were kept and euthanized in the research station of Frankenforst at University of Bonn, following German pig breeding guidelines [24]. AMs were obtained from bronchoalveolar lavage (BAL) of animals. In brief, lungs were lavaged with 200 ml ice-cold sterile calcium-magnesium free Dulbecco's phosphate-buffered saline (PBS) (pH 7.4) that was instilled gently in 25 ml aliquots into the each of two adjacent lung subsegments and withdrawn immediately. BAL fluid from each animal was collected in separate tubes and filtered through sterile gauze. Cells were centrifuged at 4°C for 10 min at 400 × g. Pellets of bronchoalveolar cells were washed twice with sterile D-PBS at 250 × g for 10 min and resuspended in 2 mM L-glutamine-containing complete RPMI-1640 media (Sigma) supplemented with 10% fetal calf serum (Invitrogen) and containing antibiotics and antimycotics (penicillin, streptomycin and amphotericin, Invitrogen). The average purity of AM cells was 91% and other cells were mostly polymorphonuclear cells (8%) and remaining was lymphocytes. The cell viability was determined by Trypan blue dye exclusion method (> 98% in all cases).

### Stimulation of alveolar macrophage cells with LPS and LTA

The cells were counted using Haemocytometer (AbCam) and concentration was adjusted. The AMs were plated in ultra-low attachment polystyrene 24-wells plate (CellStar) at 2 × 10^6 ^cells in 1 ml medium in each well. Plates were incubated at 37°C with 5% CO_2 _(Heraeus Instrument) for 48 h. After 1 h incubation, cells were stimulated with LPS of *Escherichia coli *055:B5 (Sigma) (10 μg per ml per well), LTA of *Staphylococcus aureus *(Sigma) (10 μg per ml per well) and with both of LPS and LTA (10 μg per ml per well). Cells were then collected at 1, 4, 8, 12, 24 and 48 h after stimulation for RNA extraction and stored at -80°C. For every time point non-stimulated control group was also included.

### RNA extraction and cDNA synthesis

Harvested AM cells were washed in RPMI-1640 medium and the total RNA was extracted using Pico-Pure RNA isolation kit following the manufacturer's protocol (Arcturus, Applied Biosystems). In order to remove possible contaminating genomic DNA, the extracted RNA was treated with 5 μl RQ1 DNase buffer, 5 units DNase and 40 units of RNase inhibitor in a 40 μl reaction volume. The mixture was incubated at 37°C for 1 h followed by purification with the RNeasy Mini Kit (Qiagen). Concentration of clean-up RNA was determined spectrophotometrically by using the NanoDrop ND-8000 (Thermo Scientific) instrument; the purity of RNA was estimated by the ratio A260/A280 with respect to contaminants that absorb in the UV. Additional examination of integrity was done by denaturing agarose gel electrophoresis and ethidium bromide staining. Finally, the purified RNA was stored at -80°C for further analysis. Approximately 1.5 μg of total RNA for each sample was transcribed into cDNA. cDNA was synthesised with SuperScript-II RT kit (Invitrogen). All samples were reverse transcribed under the same conditions. The synthesized cDNA was stored at -20°C and used in qRT-PCR reactions as a template.

### Selection of reference genes and primer design

There are few previous studies for validation of selected HKGs across various tissues in pigs [11,12,16,25,26] with specific purpose and no study was devoted to validate reference genes in the AMs in case of inflammatory disease condition or in response to the bacterial product LPS and/or LTA. However, 'traditional' reference genes like *GAPDH *and *TBP *have been most often used in pigs [12,16,27-32]. Regarding porcine organs, *ACTB, B2M, GAPDH, HMBS, HPRT1, RPL4, SDHA, TBP *and *YWHAZ *have been previously compared [16,21]. More specifically in recent days, *GAPDH, ACTB, RPL27, RPS29, RPS13 *are compared in porcine stomach [31]; *GAPDH, TBP, HPRT, RPS29, ACTB *and *RPL27 *are validated in porcine adipose tissues in different breeds of pigs [26] and *B2M, SDHA, ACTB, GAPDH, HPRT1 *and *TBP *expression stability are compared in porcine muscle and liver tissues in pigs [25]. The genes used in our study were selected based on these previous studies. The following nine commonly used reference genes were selected: *ACTB, GAPDH, HPRT1, B2M, SDHA, RPL4, YWHAZ, TBP *and *PPIA *(Table 1). Primers were designed using the publicly available web-based Primer3 program [33] and are listed in Table 1. They were tested using a BLAST analysis against the NCBI database http://www.ncbi.nlm.nih.gov/tools/primer-blast.

**Table 1:** Selected candidate reference genes, primers, and PCR reactions efficiencies

Gene name	GenBank accession number	Primer sequence	Amplicon length (bp)	Amplification efficiency (%)	^a^R^2^	Average Ct of cDNA			
						
						Control	LPS	LTA	Combined
*B2M*	NM_213978.1	F:ACTTTTCACACCGCTCCAGT R:CGGATGGAACCCAGATACAT	180	89.45	0.992	25.46	24.30	23.58	23.34

*BLM*	NM_001123084.1	F:TCCTCACCTTCTGCATTTCC R:GTGGTGGCTGAGAATCCTGT	152	93.12	0.993	30.47	28.58	27.54	28.06

*GAPDH*	AF017079.1	F:ACCCAGAAGACTGTGGATGG R:ACGCCTGCTTCACCACCTTC	247	89.45	0.994	36.96	35.59	33.92	34.32

*HPRT1*	NM_001032376.2	F:AACCTTGCTTTCCTTGGTCA R:TCAAGGGCATAGCCTACCAC	150	91.88	0.997	29.21	28.16	28.50	27.89

*PPIA*	NM_214353.1	F:CACAAACGGTTCCCAGTTTT R:TGTCCACAGTCAGCAATGGT	171	91.32	0.997	23.79	22.88	23.38	23.27

*RPL4*	DQ845176.1	F:AGGAGGCTGTTCTGCTTCTG R:TCCAGGGATGTTTCTGAAGG	185	90.21	0.993	25.47	24.92	23.57	24.19

*SDHA*	DQ178128.1	F:AGAGCCTCAAGTTCGGGAAG R:CAGGAGATCCAAGGCAAAAT	149	92.24	0.996	30.35	29.28	28.21	28.11

*TBP*	DQ178129.1	F:ACGTTCGGTTTAGGTTGCAG R:GCAGCACAGTACGAGCAACT	118	99.43	0.997	31.81	30.59	31.11	30.25

*YWHAZ*	DQ178130.1	F:ATTGGGTCTGGCCCTTAACT R:GCGTGCTGTCTTTGTATGACTC	146	94.52	0.994	24.50	23.74	23.78	23.23

^a ^R^2^, correlation coefficient calculated from slope of the standard curve

### Quantitative real-time PCR (qRT-PCR)

**Figure 1:**
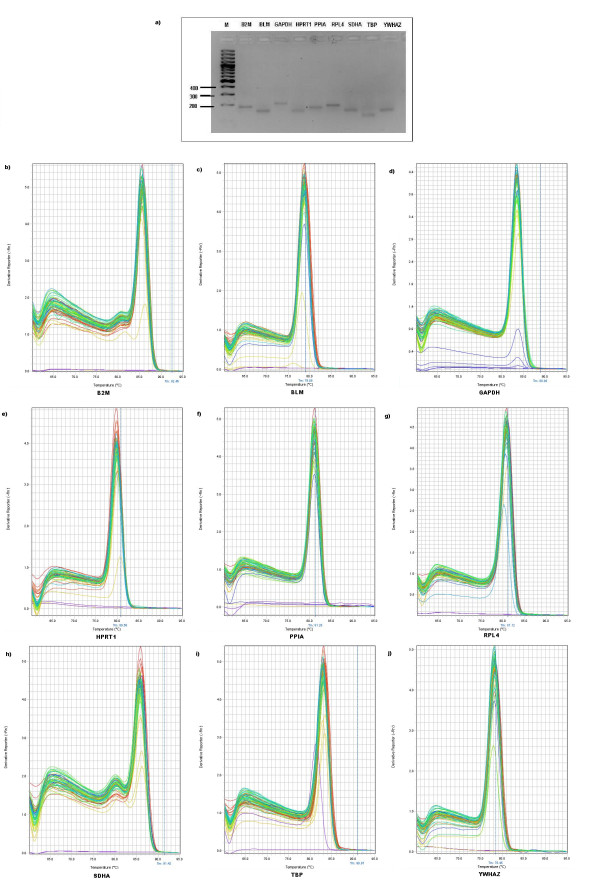
**Confirmation of amplicon size and primer specificity of studied genes**. **a**) Agarose gel electrophoresis showing specific reverse transcription PCR products of the expected size for each gene, M represents DNA size marker. **b**) Melting curve analysis for all amplicons.

Nine-fold serial dilution of plasmids DNA were prepared and used as template for the generation of the standard curve. In each run, the 96-well microtiter plate contained each cDNA sample, plasmid standards for the standard curves and no-template control. A no-template control (NTC) was included in each run for each gene to check for contamination. Quantitative real-time RT-PCR (qRT-PCR) was set up using 2 μl first-strand cDNA template, 7.4 μl deionized H_2_O, 0.3 μM of upstream and downstream primers and 10 μl 1× Power SYBR Green I master mix with ROX as reference dye (Bio-Rad). The thermal cycling conditions were 3 min at 95°C followed by 15 s at 95°C (40 cycles) and 1 min at 60°C. Experiments were performed using the StepOnePlus™ Real-Time PCR System (Applied Biosystems). Based on the Ct-values for all dilution points in a series, a standard curve was generated using linear regression and the PCR amplification efficiency of each primer pair is calculated from the slope of a standard curve [15]. Melting curve analysis was performed to verify the presence of gene-specific peaks and the absence of primer dimmers (Figure 1b). Agarose gel electrophoresis was performed to test for the specificity of the amplicons (Figure 1a). To ensure repeatability of the experiments, all the reactions were executed in duplicate and the mean was used for further analysis.

### Determination of reference gene expression stability

The raw qRT-PCR amplification data was exported from the StepOne^® ^software (Applied Biosystem) to Microsoft^® ^Excel. The averages of the Ct-values for each duplicate were used for stability comparison of candidate reference genes in the NormFinder, geNorm and BestKeeper software. For easy understanding, the samples were grouped into 5 different categories such as LPS stimulated, LTA stimulated, LPS + LTA (combined), control and irrespective to stimulation group (when all the stimulated and non-stimulated control were considered together). The effect of stimulation and time on the expression of housekeeping genes was tested using GLM procedure of the SAS software (ver.9.2; SAS, SAS Institute Inc., Cary, NC, USA). Differences in gene expression levels between time and stimulation were determined using *t*-test in SAS. *P *< 0.05 was considered statistically significant.

Ct-values of all samples were exported to Excel, ordered for use in geNormPlus software (15 days free trial version qBasePlus; http://www.biogazelle.com) and geNorm transformed to relative quantities using the gene-specific PCR amplification efficiency [34]. These relative quantities were then exported to geNormPlus to analyze gene expression stability [8]. The approach of reference gene selection implemented in geNorm relies on the principle that the expression ratio of two ideal reference genes should be identical in all samples, independent of the treatment, condition, or tissue type. Increasing variations in the expression ratio between two genes correspond to lower expression stability across samples. geNorm calculates the stability using a pairwise comparison model [8]. geNorm determines the level of pairwise variation for each reference gene with all other reference genes as the standard deviation of the logarithmically transformed expression ratios. In this way, the reference gene expression stability measure (*M *value) was calculated as the average pairwise variation of a particular gene with all other control genes included in the analysis [8,15]. Lower *M *values represent higher expression stabilities. Sequential elimination of the least stable gene (highest *M *value) generates a ranking of genes according to their *M *values and results in the identification of the genes with the most stable expression in the samples under analysis. geNorm was also used to estimate the normalization factor (NF_*n*_) using *n *multiple reference genes, by calculating the geometric mean of the expression levels of the *n *best reference genes [8]. The optimisation of the number of reference genes starts with the inclusion of the two genes with the lowest *M *value, and continues by sequentially adding genes with increasing values of *M*. Thus, geNorm calculates the pairwise variation V_*n*_/V_*n*+1 _between two sequential normalization factors NF_*n *_and NF_*n*+1 _containing an increasing number of reference genes [8]. A large variation means that the added gene has a significant effect on the normalization and should preferably be included for calculation of a reliable normalization factor. Ideally, extra reference genes are included until the variation V_*n*_/V_*n*+1 _drops below a given threshold. According to geNorm, if V_n/n+1 _< 0.15 the inclusion of an additional reference gene is not required and the recommended number of reference genes is given by *n *[8].

NormFinder uses an ANOVA-based model [35]. The software calculates a stability value for all candidate reference genes tested. The stability value is based on the combined estimate of intra- and inter-group expression variations of the genes studied [35]. For each gene, the average Ct value of each duplicate reaction was converted to relative quantity data as described for geNorm, to calculate the stability value with NormFinder program [35]. The NormFinder reference tool was applied to rank the candidate reference gene expression stability for all samples with no subgroup determination (irrespective to stimulation) as well as with stimulation (LPS, LTA, and both LPS and LTA) as subgroup. A low stability value, indicating a low combined intra- and inter-group variation, indicates high expression stability [35].

The average Ct-value of each duplicate reaction was used (without conversion to relative quantity) in BestKeeper to analyze the stability value of studied genes [36]. BestKeeper creates a pairwise correlation coefficient between each gene and the BestKeeper index (BI). This index is the geometric mean of the Ct-values of all candidate reference genes grouped together. BestKeeper also calculates standard deviation (SD) of the Ct-values between the whole data set. The gene with the highest coefficient of correlation with the BI indicates the highest stability [36].

## Results

### Purity, quantity of extracted RNA and verification of amplicons

The optical density (OD) ratio A260/A280 nm measured with a Nanodrop spectrophotometer was 1.94 ± 0.17 (OD A260/A280 ratio ± SD). The average RNA concentration after extraction using Pico Pure was 10.33 μg/μl ± 1.1 (μg/μl ± SD). The results of the averaged amplification efficiencies are shown in Table 1. The amplification efficiencies for the nine candidate reference genes ranged between 89.45% and 99.43%. The agarose gel electrophoresis (Figure 1a) and melting curve analysis (Figure 1b-j and Table 1) revealed that all primer pairs amplified a single PCR product with expected size. Furthermore, sequence analysis of cloned amplicons revealed that all sequenced amplified fragments were identical to sequences used for primer design from GenBank (data not shown).

### Expression levels of candidate reference genes

**Figure 2:**
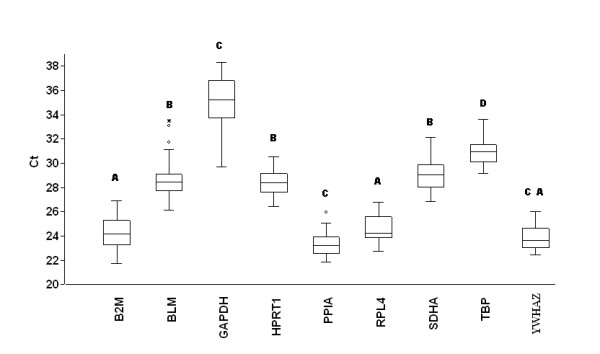
**Average cycle threshold (Ct) values of candidate reference genes tested in AMs under different conditions**. **a**) The values are the average qRT-PCR cycle threshold numbers (Ct values). The bars indicate standard deviation. Letters indicate a significant difference in average Ct value. Average Ct values that have the same letter are not significantly different (*P *> 0.05)

Transcript abundance of commonly used HKGs were analysed in the different samples by direct comparison of their cycle threshold (Ct), assuming equal Ct for equal transcript number since all qRT-PCR reactions were performed with an equal quantity of total RNA. Figure 2 showed that seven out of the nine selected genes presented Ct-values that ranged from 20 to 30 cycles, while Ct-values from *GAPDH *(mean Ct 35.20) and *TBP *(mean Ct 30.94) were lower. The Ct of the remaining selected genes showed a reasonable dispersion to moderately high expression levels. The expression *PPIA *and *YWHAZ *were followed by *B2M *(mean Ct 24.17), *RPL4 *(mean Ct 24.54), *HPRT1 *(mean Ct 28.44) and *BLM *(mean Ct 28.67). *GAPDH *expression was lowest as indicated by Ct-values around 35 cycles, but it exhibited rather high dispersion over the stimulations and culture conditions indicated by large whiskers of the box (Figure 2a). According to variance analysis, the expression of eight genes was different from each other (*P *< 0.0001) (Figure 2).

### LPS and LTA affect expression level of reference genes

**Figure 3:**
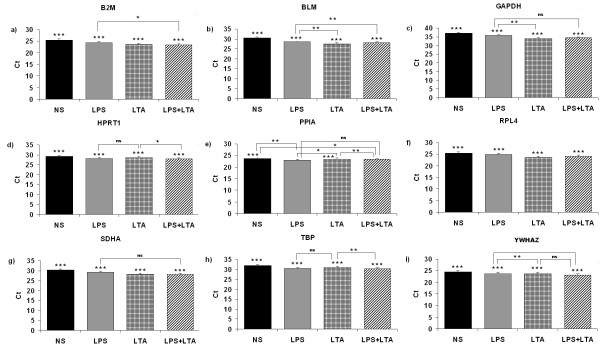
**Expression levels of a representative subset of nine HKGs**. **a**) *B2M*, **b**) *BLM*, **c**) *GAPDH*, **d**) *HPRT1*, **e**) *PPIA*, **f**) *RPL4*, **g**) *SDHA*, **h**) *TBP *and **i**) *YWHAZ*. Irrespective to stimulation: when all the stimulated and non-stimulated control were considered together; NS: no stimulation; LPS: lipopolysaccharide; LTA: lipoteichoic acid; LPS + LTA (combined): lipopolysaccharide used together with lipoteichoic acid. Differences among groups indicated with stars. *: *P *< 0.05; **: *P *< 0.01; ***: *P *< 0.001

The current study investigated fluctuations in expression of nine HKGs in AMs cultured with no stimulation, or stimulated with LPS, LTA or both. There were some fluctuations in the expression level of these genes in certain conditions. The expression differences of these genes are shown in Figure 3. The variance analysis results between treatment groups and time of stimuli to the AMs are shown in Additional file 1: Table S1. Cell harvest time significantly affected the expression level of HKGs (*P *< 0.0001) (Additional file 1: Table S1). When the no stimulation control group was compared with the stimulated groups, the expression levels of all genes were lower in non-stimulated control group (Figure 3, Table 1). With LPS stimulation, mRNA expression levels of nine genes were increased. Beside in the case of LTA stimulation, expression levels of nine genes were increased compared to control group. Only expression of PPIA was decreased when cells were stimulated with both LPS and LTA compared to LPS stimulation only (Figure 3e).

### Identification of optimal reference genes

**Figure 4:**
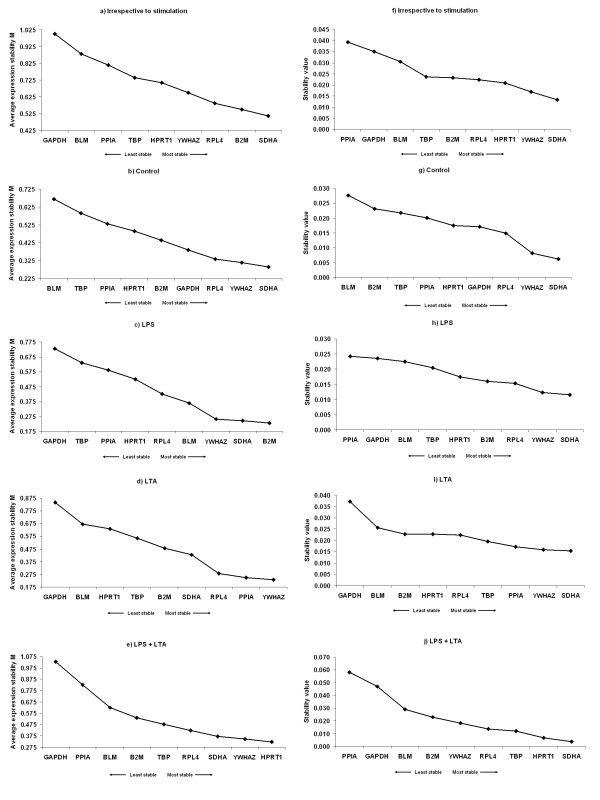
**Ranking of nine candidate reference genes using geNorm and NormFinder softwares**. (**a**-**e**) geNorm ranks the candidate reference genes based on their stability parameter M. The lower the M value, the higher the expression stability. (**f**-**j**) NormFinder ranks the genes based on a calculated stability value. The lower the stability value, the higher the expression stability. Irrespective to stimulation: when all the stimulated and non-stimulated control were considered together; Control: no stimulation; LPS: lipopolysaccharide; LTA: lipoteichoic acid; LPS + LTA (combined): lipopolysaccharide used together with lipoteichoic acid.

Transcription profiling using qRT-PCR assays was then performed with these nine candidate genes, in samples from the four different conditions of AM cultures (LPS, LTA, combined LPS and LTA, and control). These raw Ct data were then analysed using different algorithms to identify the most suitable candidate genes. In each independent culture, the 9 genes were ranked according to their gene expression stability measure "*M*" (Figure 4a-e, left panel) with using the geNorm algorithm. Stepwise exclusion of the least stable gene allowed the genes to be ranked according to their *M *value (the lower the M value, the higher the gene's expression stability) [8]. All genes presented an *M *value below 1.5, which is the default limit for acceptable expression stability as defined by Vandesompele et al. [8]. Figure 4a shows the ranking of the nine candidate reference genes across the AMs based on their stability values without considering the type of stimulation of cells i.e. irrespective of stimulation group. *SDHA, B2M *and *RPL4 *were identified as the most stable HKGs (Figure 4a) in the irrespective of stimulation group. In case of the control group, geNorm showed that *SDHA, B2M *and *RPL4 *were the most stable HKGs (Figure 4b). When AMs were stimulated with Gram negative bacterial product LPS, geNorm identified *B2M, SDHA *and *YWHAZ *as the most stable HKGs (Figure 4c). *YWHAZ, PPIA *and *RPL4 *were the most stably expressed HKGs in the case of Gram-positive bacterial product (LTA) stimulation group (Figure 4d). When LPS was used combined with LTA for the stimulation of AMs, *HPRT1, YWHAZ *and *SDHA *remained the most stable genes (Figure 4e). All investigated groups identified *GAPDH *as the least stable reference gene by geNorm (Figure 4a, c, d and 4e) except in control group where *BLM *was the least stable HKG (Figure 4b).

NormFinder software ranked all HKGs according to their stability value (Figure 4f-j) [35]. The expression stability was not always consistent between the used softwares. By using NormFinder, genes *SDHA, YWHAZ *and *HPRT1 *were ranked as the most stable HKGs in irrespective to stimulation group (Figure 4f). In the non-stimulated control group and LPS stimulated group, *SDHA, YWHAZ *and *RPL4 *remained the most stable genes (Figure 4g-h). In the LTA stimulated group, *SDHA, YWHAZ *and *PPIA *were ranked as the most stable HKGs (Figure 4i). In the combined LPS and LTA stimulation group, *SDHA, HPRT1 *and *TBP *were found to be most stable HKGs (Figure 4j). *PPIA *remained the least stable HKGs followed by *GAPDH *and *BLM *according to the NormFinder algorithm.

The results of reference gene evaluation by the BestKeeper tool are shown in Table 2. According to the variability observed, candidate reference genes can be identified as the most stable genes, as they exhibited the lowest coefficient of variance (CV ± SD). A low SD of the cycle threshold (Ct) values should be expected for a useful reference gene. It is important to note that, genes that show a SD higher than 1 should be considered as unacceptable [36,37]. In the irrespective to stimulation group, *YWHAZ *was identified as the most stable HKG whereas *GAPDH, BLM *and *B2M *were removed from the initial statistics (Table 2). In the control group, PPIA was shown to be the most stable HKG by BestKeeper. *SDHA, BLM *and *RPL4 *was identified as the most stable HKG by NormFinder in the case of LPS, LTA stimulated and combined LPS and LTA stimulated group, respectively (Table 2); whereas in all these cases, only *GAPDH *was eliminated from the initial statistics.

**Table 2:** Expression stability of nine candidate reference genes evaluated by BestKeeper software

	*B2M*	*BLM*	*GAPDH*	*HPRT1*	*PPIA*	*RPL4*	*SDHA*	*TBP*	*YWHAZ*	BI
*Irrespective to stimulation*										

n	48	48	48	48	48	48	48	48	48	48

SD [± Ct]	1.05	1.10	1.61	0.78	0.79	0.89	1.03	0.81	0.76	0.83

CV [% Ct]	4.36	3.84	4.57	2.73	3.37	3.64	3.57	2.63	3.20	3.04

*Control*										

n	12	12	12	12	12	12	12	12	12	12

SD [± Ct]	0.99	1.33	1.07	0.93	0.63	1.07	1.00	0.74	0.82	0.93

CV [% Ct]	3.88	4.35	2.91	3.19	2.65	4.18	3.30	2.34	3.34	3.27

*LPS*										

n	12	12	12	12	12	12	12	12	12	12

SD [± Ct]	0.46	0.73	1.25	0.61	0.60	0.75	0.44	0.73	0.56	0.59

CV [% Ct]	1.89	3.52	2.54	2.16	2.60	3.00	1.52	2.39	2.35	2.16

*LTA*										

n	12	12	12	12	12	12	12	12	12	12

SD [± Ct]	0.95	0.42	1.53	0.61	0.75	0.73	0.91	0.70	0.72	0.74

CV [% Ct]	4.04	4.51	1.53	2.15	3.19	3.11	3.23	2.27	3.04	2.75

*LTA + LPS*										

n	12	12	12	12	12	12	12	12	12	12

SD [± Ct]	0.59	0.39	1.33	0.46	0.98	0.36	0.39	0.49	0.53	0.34

CV [% Ct]	2.52	1.37	3.89	1.66	4.21	1.49	1.38	1.62	2.30	1.26

Descriptive statistics of nine candidate reference genes based on their cycle threshold (Ct) values. In the last column the BestKeeper (BI) index is computed together with the same descriptive parameters for nine genes. *Abbreviations: n *number of observations, *CV [%Ct] *the coefficient of variance expressed as a percentage on the Ct level, *SD [± Ct] *the standard deviation of the Ct. Results from overall tissues irrespective of stimulation, non-stimulated control and different stimulations (LPS, LTA and LPS + LTA) are shown

### Determination of the optimal number of reference genes for normalization

**Figure 5:**
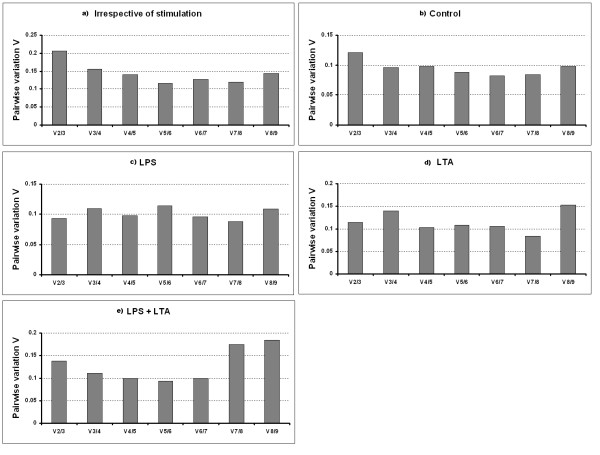
**Determination of the optimal number of reference genes for normalization**. The geNorm software calculates the normalization factor from an increasing number of genes (starting with at least two) for which the variable V defines the pairwise variation between two sequential normalization factors. The lower the pairwise variation, the better is the combination of genes for reference. V5/6 for example, shows the variation between the normalization factors of five genes in relation to six genes and shows that six genes is the combination providing the lowest pairwise variation. Irrespective to stimulation: when all the stimulated and non-stimulated control were considered together; Control: no stimulation; LPS: lipopolysaccharide; LTA: lipoteichoic acid; LPS + LTA (combined): lipopolysaccharide used together with lipoteichoic acid.

**Figure 6:**
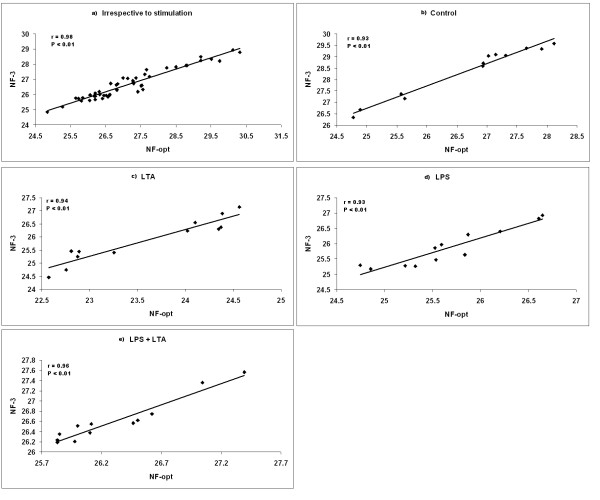
**Correlation between the NF of most three stable and optimal number endogenous control**. Pearson's correlations between the NFs of three endogenous control genes (NF3) and optimal number (six) of endogenous control genes (NFopt) for **a**) all samples irrespective of stimulation, **b**) non-stimulated control, **c**) LTA stimulated AMs, **d**) LPS stimulated AMs and e) LPS and LTA together used for stimulation. Irrespective to stimulation: when all the stimulated and non-stimulated control were considered together; Control: no stimulation; LPS: lipopolysaccharide; LTA: lipoteichoic acid; LPS + LTA (combined): lipopolysaccharide used together with lipoteichoic acid.

The geNorm program calculates the normalization factor assessing the optimal number of reference genes for generating the *M *factor by calculating the pair-wise variation *V*. The pair-wise variation between these genes defines the variable *V*. The lower the variable *V *is, the less variation. The overall results are shown in Figure 5. For the irrespective to stimulation and combined LPS and LTA groups as shown in Figure 5a and 5e, five endogenous control genes are necessary to obtain the lowest changing *V *values in the analysed samples. On the other hand, seven endogenous HKGs were required for both the LPS and LTA stimulated groups (Figure 5c and 5d). For the control group, six HKGs were required to obtain an accurate normalization factor (Figure 5b). However, it is impractical to use excessive numbers of endogenous control genes for normalization, particularly when only a small number of target genes need to be studied or for rare samples that are very difficult to acquire [8,12]. Therefore, the use of the three most stable HKGs for the calculation of the NF was considered acceptable for the majority of experiments [8,12]. To verify that the use of three HKGs simultaneously is adequate for normalization of qRT-PCR data, the correlation of NF values between the geometric means of the three most stable genes and the optimal number of genes was calculated for all sample groups. As shown in Figure 6, there is a high correlation between the two NF measures (i.e., the theoretical optimal number and proposed number, three) for all groups including irrespective to stimulation group (*r *= 0.93-0.98, Pearson) (Figure 6a-e). This result demonstrates that the three most stable HKGs are sufficient for an accurate normalization of qRT-PCR data [8,12].

## Discussion

Using reference genes that have a stable expression between the compared groups is crucial in gene expression studies. Several studies have shown that the use of different reference genes can change the outcome and conclusions of a study [13,19,38]. Ideally, the internal control gene for quantitative gene expression studies should not be influenced by the conditions of the experiment. However, our study showed that expression of the HKGs was affected by stimulation type as well as stimulation duration (Additional file 1: Table S1). Therefore it is generally recommended that the stability of HKGs is being validated prior to expression studies. There are some reports of the expression levels of HKGs in various cells and tissues and also of the methods used to analyse the stability of these genes. Recent research has demonstrated that the expression of HKGs may be altered due to state of the organ [21,39], age [17,21,26] and experimental conditions [18,20,40]. In the characterization of the course of an inflammatory reaction, quantitative real-time PCR has become a powerful tool for detection of inflammatory parameters, including cytokines and Toll-like receptors (TLRs). This tool is particularly useful in pigs since commercial species-specific antibodies directed against pig cytokines and TLRs are not commonly available. To best of our knowledge, there has not yet been a detailed evaluation of HKGs in swine AMs. Moreover, there has not been a detailed study under different types of stimulation such as LPS, LTA and combined LPS and LTA that might be indicated Gram-negative, Gram-positive bacterial infection or co-infection of both types of bacteria *in vivo*. Although no in depth studies are apparent in the AMs cells, there have been numerous research papers which have used single HKGs for normalisation of gene expression in AMs. These have included the use of *HPRT1 *[5], *GAPDH *[41] and *18S *rRNA [7] for normalisation of gene expression. As a consequence, in this study, we evaluated the gene expression stability of nine commonly used HKGs in porcine AMs, and furthermore, assessed their stability in states of different inflammatory models such as in response to LPS and LTA.

In recent years, there have been a number of research papers and reviews evaluating the selection and effect of controls on normalised gene expression data in various pig tissues. Gu et al. [12] involved in the validation of 20 common endogenous control genes in 56 fat- and muscle-type tissues. Nygard et al. [16] investigated a vast number of tissues for 10 HKGs. Studies focusing on more specific tissues, including the backfat, longissimus dorsi muscle [11], liver [25], adipose [26], stomach [31] and mesenchymal stem cells [42] are being reported in pigs. Taken together, it is very difficult to find a 'universal' reference gene having stable expression in all cell types and tissues, and in particular to find reference genes that remain stable under different experimental or infectious conditions. According to the NCBI-PubMed statistics [12], *GAPDH *and *ACTB *are the two mostly used porcine HKGs. But they have been shown to vary considerably and are consequently unsuitable as reference genes for normalization of gene expression analysis in many cases [43-45]. We applied three software programs to our data as complementary analyses to obtain the most suitable genes for our experiments. Both algorithms resulted in an overall comparable order of genes. Two of the three best genes were always presented by geNorm and NormFinder. Although BestKeeper [36] is found on the same principle as geNorm, not in every case both algorithms displayed overlapping suitable HKGs.

In the present study, geNorm and NormFinder showed that *SDHA, YWHAZ *and *RPL4 *are the most stable three HKGs in the control (without any stimulation) group as well as in stimulation groups (Figure 4). Our results are in good agreement with Piórkowska et al. [26] who identified *GAPDH *and *TBP *as the least stable HKGs for the porcine adipose tissue. Beside, *TBP *was always found to be as a moderately stably expressed gene in this study. Nygard et al. [16] reported that *RPL4, TBP *and *YWHAZ *have the highest stability across tissues collected from healthy pigs which are somewhat consistent with the present study. Pierzchala et al. [25] recently reported that *HPRT1 *and *TBP *are the most stable HKGs in porcine liver and in different skeletal muscle tissues but it could be found that *HPRT1 *and *TBP *is moderately stable through different experiments conditions in this study (Figure 4). Moreover, Svobodová et al. [46] estimated *HPRT1 *has the highest stability while *GAPDH *was unstable across different porcine tissues which are in agreement with our result for the *GAPDH *but not for *HPRT1*. Because *HPRT1 *was found to be moderately stable in our experiment, except in combined LPS and LTA group.

To our knowledge, there are only two studies evaluating the stability of reference genes in AMs. One being in human AMs [1] and the other being in the horse [10]; no data is available on the stability of reference genes in AMs of other mammalian species. Ishii et al. [1] reported that *HPRT1 *is the most stable HKG, whereas *TBP *is the least stable HKG in both the LPS stimulated and non-stimulated AMs in human which is in good agreement with our result using geNorm. (Figure 4b-c). Beekman et al. [10] used geNorm to validate the candidate HKGs and found that *GAPDH, SDHA, HPRT *and *RPL32 *were the most stably expressed genes in bronchoalveolar lavage cells of horses with inflammatory airway disease with corticosteroids treatment. In this study, *SDHA *was identified as suitable reference gene by using NormFinder through the experiments which is agreement with the report in horse [10].

According to the BestKeeper analysis software, in the irrespective to stimulation group *YWHAZ *was detected in accordance with the NormFinder and partially with the geNorm results (Table 2; Figure 4). *SDHA *was identified as the most stable gene in both geNorm and NormFinder (Figure 4); however, BestKeeper identified this gene as unsuitable according to its algorithm criteria. In the control group, although *PPIA *was identified as a stably expressed HKG by BestKeeper (Table 2), this gene was identified moderately stable in geNorm and NormFinder (Figure 4). By using the three software algorithms similar results were obtained in LPS stimulated group, where *SDHA *was identified as the most stable HKG. In the LTA stimulated group, although *BLM *was identified as the most stable HKG by BestKeeper, but showed very low expression stability in geNorm and NormFinder. In case of the combined LPS and LTA stimulated group, *RPL4 *was found to be the most stable gene by BestKeeper (Table 2); however, this gene ranked as the fourth most stable HKG by geNorm (Figure 4). Several studies previously reported similar discrepancies for the findings of BestKeeper [15,31,37] and importantly, few studies followed the BestKeeper analysis method compared to geNorm and NormFinder. It is important to note that very similar discrepancies between the different algorithms have been observed in previous studies comparing statistical analysis methods [10,15,31,37,47].

However, we found that the first three most stable reference genes in most cases were consistent between the software geNorm and NormFinder, even if they were not in the exact same ranking order. Similar findings are reported by previous studies in horse, human and plants [10,13,15,47]. Such discrepancy could be explained by genes' co-regulation. Indeed, co-regulated genes may become highly ranked independently of their expression stabilities with geNorm software [35]. Moreover, NormFinder takes into account variation across subgroups, thus avoiding artificial selection of co-regulated genes by analyzing the expression stability of candidate genes independently from each other [8]. However, no studies dealing with porcine reference genes stability used other analysis methods except geNorm [11,12,16,26,31,42].

As described above, geNorm also provides a measure for the best number of reference genes that should be used for optimal normalization. In agreement with several previous studies, we postulate that the use of more than one reference gene allows for a more accurate normalization than the use of only one reference gene [8,12,35]. Based on a cut-off point for the *V *value, as described by Vandesompele et al. [8], a combination of the several most stable reference genes was calculated as being optimal for gene expression studies in control and PAMPs stimulated porcine AMs (Figure 5). However, as we described above and other studies [8,12] recommended, the combination of the most three stable genes are appropriate for accurate normalization.

## Conclusions

In conclusion, this investigation found evidence that there can be variation in the expression of commonly used HKGs due to different PAMPs. Due to the new influx of data suggesting alterations in mRNA expression according to bacteria type, we feel that beside therapy uses or experimental condition, there needs to be special consideration given to the selection of HKGs based upon the bacterial pathogen identification. This indicates that the choice of reference genes cannot be transposed from on study to the other without validation for the specifics of each experimental protocol. Since different bacterial pathogens are cooperating in the respiratory tract as co-infection, our results will shed light on pathogenic or disease status of experiments. In general, we recommend using the geometric mean of *SDHA, B2M *and *RPL4 *to guarantee suitable normalization in across the AMs with unknown respiratory pathogenic condition in pigs. Since in the most cases, Gram-negative and Gram-positive bacteria are observed together in respiratory diseases, *HPRT1, YWHAZ *and *SDHA *might be an appropriate set of reference genes for the gene expression normalization in AM studies. *SDHA, YWHAZ *and *RPL4 *could be suggested in case of AMs without any stimulation. This study offers an appropriate set of HKGs that might be used in the normalization of gene expression data *in vitro *cultured porcine AMs.

## Abbreviations

qRT-PCR: quantitative real-time reverse transcriptase polymerase chain reaction; *B2M*: Beta-2-microglobulin; *BLM*: Bloom syndrome: RecQ helicase-like; *GAPDH*: Glyceraldehyde 3-phosphate dehydrogenase; *HPRT1*: Hypoxanthine phosphoribosyltransferase 1; *PPIA*: Peptidylprolyl isomerase A (cyclophilin A); *RPL4*: Ribosomal protein L4; *SDHA*: Succinate dehydrogenase complex subunit A flavoprotein; *TBP*: TATA box binding protein; *YWHAZ*: Tyrosine 3/tryptophan 5-monooxygenase activation protein zeta polypeptide; NTC: No-template control; Ct: Cycle threshold; SD: Standard deviation; BI: BestKeeper Index; HKG: Housekeeping gene

## Competing interests

The authors declare that they have no competing interests.

## Authors' contributions

MUC performed the qPCR experiments, analysed data and prepared and edited the manuscript. MAI isolated AMs and made *in vitro *experiments. MJU analysed data and prepared the manuscript with MUC. DT, ET and CL edited the manuscript. KS criticized the experimental design and edited the manuscript. All authors read and approved the final manuscript.

## Supplementary Material

Additional file 1 **Table S1**. Relative expression of candidate genes and effect of treatment and time of stimuli on mRNA expression level. Overall expression data of reference candidate genes. Summary of the Proc GLM (ver.9.2; SAS, SAS Institute Inc., Cary, NC, USA) analysis detecting effect of stimulation type, duration of stimulation *in vitro*, duration and stimulation type interaction on the mRNA expression of reference candidate genes. Click here for file
