# Supplementary material for: Evaluation of suitable reference genes for gene expression studies in porcine alveolar macrophages in response to LPS and LTA
Source: BMC Res Notes. 2012 Feb 18;5:107. doi: 10.1186/1756-0500-5-107 (PMC3306271; doi:10.1186/1756-0500-5-107)
Supplement: Additional file 1 — Table S1. Relative expression of candidate genes and effect of treatment and time of stimuli on mRNA expression level. Overall expression data of reference candidate genes. Summary of the Proc GLM (ver.9.2; SAS, SAS Institute Inc., Cary, NC, USA) analysis detecting effect of stimulation type, duration of stimulation in vitro, duration and stimulation type interaction on the mRNA expression of reference candidate genes. [file 1756-0500-5-107-S1.DOC]

**Table S1 Relative expression of candidate genes and effect of treatment and time on expression level**

**(calculated by PROC GLM, SAS)**

|  | Mean±SD | Treatment | Time | Treatment*Time | R^2^ | Model |
| --- | --- | --- | --- | --- | --- | --- |
| B2M | 24.17±1.3 | <0.0001 | <0.0001 | <0.0001 | 0.989 | <0.0001 |
| BLM | 28.66±1.5 | <0.0001 | <0.0001 | <0.0001 | 0.978 | <0.0001 |
| GAPDH | 35.20±2.0 | <0.0001 | <0.0001 | <0.0001 | 0.952 | <0.0001 |
| HPRT1 | 28.44±0.9 | <0.0001 | <0.0001 | <0.0001 | 0.957 | <0.0001 |
| PPIA | 23.33±1.0 | <0.0001 | 0.0004 | <0.0001 | 0.946 | <0.0001 |
| RPL4 | 24.54±1.1 | <0.0001 | <0.0001 | <0.0001 | 0.988 | <0.0001 |
| SDHA | 28.99±1.3 | <0.0001 | <0.0001 | <0.0001 | 0.986 | <0.0001 |
| TBP | 30.94±1.1 | <0.0001 | <0.0001 | <0.0001 | 0.994 | <0.0001 |
| YWHAZ | 23.81±0.9 | <0.0001 | <0.0001 | <0.0001 | 0.939 | <0.0001 |
